# Higher Serum 25-Hydroxyvitamin D Is Associated with Lower All-Cause and Cardiovascular Mortality among US Adults with Nonalcoholic Fatty Liver Disease

**DOI:** 10.3390/nu14194013

**Published:** 2022-09-27

**Authors:** Yuxiong Chen, Siqin Feng, Zhen’ge Chang, Yakun Zhao, Yanbo Liu, Jia Fu, Yijie Liu, Siqi Tang, Yitao Han, Shuyang Zhang, Zhongjie Fan

**Affiliations:** Department of Cardiology, Peking Union Medical College Hospital, Peking Union Medical College & Chinese Academy of Medical Sciences, No.1 Shuaifuyuan Wangfujing Dongcheng District, Beijing 100730, China

**Keywords:** vitamin D, death, cardiovascular, NHANES

## Abstract

Aims: We aimed to assess the association between serum 25-hydroxyvitamin D (25(OH)D) levels with all-cause and cardiovascular mortality in patients with nonalcoholic fatty liver disease (NAFLD). Methods: We performed a retrospective cohort study based on the US National Health and Nutrition Examination Survey 2001–2016 on adults aged ≥20 years. NAFLD was determined as a US Fatty Liver Index score ≥ 30 in the absence of other liver conditions. Weighted Cox proportional hazards regression models were applied to explore the relationship between serum 25(OH)D levels and mortality. Results: 898 all-cause deaths and 305 cardiovascular deaths were recorded over a median follow-up of 8.7 years. Compared with those in the severe deficiency group (below 25.0 nmol/L), the fully adjusted HRs and 95% CIs of NAFLD patients with sufficient serum 25(OH)D concentrations (≥75.0 nmol/L) were 0.36 (0.22, 0.60) for all-cause mortality and 0.14 (0.07, 0.29) for cardiovascular mortality. Each one-unit increase in the natural log-transformed serum 25(OH)D concentration was related to a 41% lower risk for all-cause deaths (HR = 0.59, 95% CI: 0.46, 0.77) and a 65% lower risk for cardiovascular deaths (HR = 0.35, 95% CI: 0.22, 0.58). Conclusions: Among NAFLD patients, increased serum 25(OH)D levels were independently associated with reduced risk for all-cause and cardiovascular deaths.

## 1. Introduction

As the first etiology of chronic liver diseases, nonalcoholic fatty liver disease (NAFLD) affected 25% of adults worldwide and 24% of adults in North America [1]. A US study has reported a growing prevalence of NAFLD from 29.5% in 1999–2000 to 40.3% in 2015–2016 [2]. As a hepatic component of metabolic dysfunction, NAFLD is strongly related to various cardiometabolic disorders, such as obesity, diabetes, and metabolic syndrome [3]. NAFLD has been proven to be related to increased mortality, mainly from cardiovascular diseases (CVD) [4]. Alvarez et al. estimated that around 8% of all-cause mortality was related to NAFLD in the US [5]. Given the growing burden of NAFLD, identifying modifiable risk factors for the prevention of mortality from NAFLD is of great urgency.

Vitamin D, a member of the steroid hormone family, mainly originates from the skin after ultraviolet radiation exposure and a small portion from food or supplements [6]. As one of the vital nutrients to human health, vitamin D takes part in many immune-inflammatory and metabolic pathways [7]. 25-hydroxyvitamin D (25(OH)D) is the established indicator of vitamin D status. Observational studies have linked decreased serum 25(OH)D levels with the progression and severity of NAFLD, whereas evidence from randomized clinical trials has found conflicting results regarding vitamin D supplementation and NAFLD [8].

Moreover, several epidemiological studies have observed that serum 25(OH)D levels were inversely related to risks of death among the general populations [9], as well as individuals with diabetes [10], hypertension [11], metabolic syndromes [12], and CVD [13]. Nevertheless, there is scarce evidence on serum 25(OH)D levels and survival among NAFLD patients. To provide evidence in this field, we assessed the impact of serum 25(OH)D levels on mortality in adults with NAFLD.

## 2. Material and Methods

### 2.1. Study Participants

**Figure 1:**
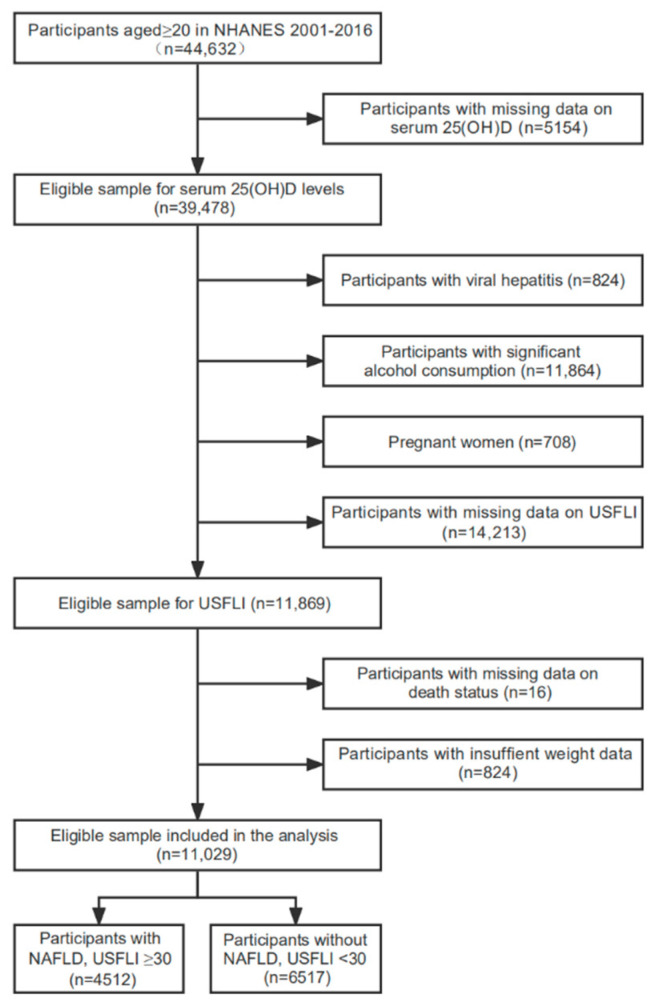
Flowchart of participant selection. Abbreviations: NAFLD, nonalcoholic fatty liver disease; NHANES, National Health, and Nutrition Examination Survey; USFLI, US fatty liver index.

The 8 continuous 2-year surveys of the National Health and Nutrition Examination Survey (NHANES) from 2001 to 2006 were used in this retrospective cohort study. Of the 44,632 participants aged ≥20 years enrolled initially, 39,478 individuals had data on serum 25(OH)D levels. Among these, we restricted our analysis to 11,869 nonpregnant participants according to the definition of NAFLD described in the section below. After excluding participants who were lost to follow-up or who had insufficient data on sample weights, 4512 individuals with NAFLD and 6517 individuals without NAFLD were included in the final analysis (Figure 1). The Ethics Review Board of the Centers for Disease Control and Prevention approved the protocols, and all individuals completed informed consent before enrollment.

### 2.2. Definition of NAFLD

We used the US fatty liver index (USFLI) to define NAFLD for the multiethnic US population, a simple non-invasive marker for the clinical diagnosis of NAFLD because of unavailable data on ultrasonography or liver biopsy. The USFLI was calculated based on age, race-ethnicity, waist circumference, γ-glutamyltransferase, fasting insulin, and fasting glucose [2,14]. Owing to several changes in the laboratory methods of fasting insulin and glucose among survey cycles, laboratory measures in later cycles were calibrated to the 2001–2002 levels by stepwise “backward” regression equations in analytic notes recommended by NCHS (Supplementary Tables S1 and S2). A USFLI score of ≥30 has been validated with high sensitivity (62%) and specificity (88%) in the US population [14].

In this study, nonpregnant individuals with USFLI values ≥30 without other known causes of chronic liver diseases, including viral hepatitis (hepatitis B surface antigen (+) or hepatitis C antibody (+)) and excessive alcohol drinking (over 2 drinks/day for men or over one drink/day for women in the past 12 months) were diagnosed as NAFLD.

### 2.3. Serum 25(OH)D Measurement

Standardized liquid chromatography-tandem mass spectrometry method was applied to measure serum 25(OH)D concentrations since the NHANES 2007–2008 survey cycle, whereas in the previous NHANES cycles, 25(OH)D concentrations were detected by the DiaSorin RIA kit (Stillwater MN). With reference to the analytical note, serum 25(OH)D values detected before 2007 were converted using ordinary least square regression equations to apply the liquid chromatography-tandem mass spectrometry-equivalent data for the analysis [15].

### 2.4. Ascertainment of Mortality

Mortality status was determined based on the National Death Index through 31 December 2019. Cardiovascular mortality was defined as deaths from heart diseases (I00–I09, I11, I13, and I20–I51) and cerebrovascular diseases (I60–I69) based on the International Classification of Disease, 10th Revision. The follow-up time covers the period between a visit to the NHANES Mobile Examination Center (MEC) and death or the end of follow-up (31 December 2019) as appropriate.

### 2.5. Study Covariates

Data on demographic information, lifestyle factors, previous medical history, and medication use were obtained through standardized questionnaires during in-home interviews by trained interviewers. Heights, weights, waist circumferences, blood pressures, and blood samples were collected from physical examinations at MEC using standard protocols.

Race/ethnicity was categorized as non-Hispanic White, non-Hispanic Black, Mexican American, and other races. Smoking status was categorized as never (under 100 cigarettes in their lifetime), former (more than one hundred cigarettes in their lifetime but quit smoking now), and current (more than one hundred cigarettes in their lifetime and still smoking now). Education levels were classified as under high school, high school or equivalent, and college or higher. The family poverty income ratio (PIR) was categorized as ≤1.0, 1.0–3.0, and >3.0. Physical activity was measured as the weekly minutes of moderate and vigorous activities multiplied by the metabolic equivalent (MET) level and divided into four categories: sedentary (without regular physical activity, MET-minutes/week = 0), insufficient (0 < MET-minutes/week < 500), moderate (500 ≤ MET-minutes/week ≤ 1000), and high (>1000 MET-minutes/week) [16]. Hypertension was diagnosed as a self-reported medical history, receiving antihypertensive drugs, or blood pressure measurement ≥ 140/90 mmHg [17]. Diabetes was diagnosed as a self-reported medical history, receiving oral hypoglycemic drugs or insulin, fasting glucose level ≥ 126 mg/dL, or hemoglobin A1c level ≥ 6.5% [18]. Hyperlipidemia was diagnosed as serum triglycerides ≥ 150 mg/L, total cholesterol ≥ 200 mg/dL, LDL-cholesterol ≥ 130 mg/dL, HDL-cholesterol ≤ 40 mg/dL in men or ≤50 mg/dL in women, or receiving medication for hyperlipidemia. The history of CVD was determined by a series of self-reported diseases (coronary heart disease, heart attack, angina, heart failure, and stroke). Steroid users were defined by reporting the use of at least one of the following medications within the past month: prednisone, prednisolone, methylprednisolone, or methylprednisolone acetate [19].

The biochemical parameters, including fasting glucose, insulin, γ-glutamyltransferase, hemoglobin A1c, blood lipids, and creatinine, were measured among partial participants providing blood samples at MEC. The estimated glomerular filtration rate (eGFR) was computed by the Chronic Kidney Disease Epidemiology Collaboration creatinine equation [20].

## 3. Statistical Analysis

Based on the NHANES analytic notes, sample weights, stratification, and clustering were considered for generalization to the whole US population aged ≥ 20 years. We divided the fasting subsample 2-year MEC weight by 8 to derive appropriate sampling weights for the combined analysis of eight survey cycles. We categorized serum 25(OH)D concentrations into 4 categories: <25.0 nmol/L, 25.0–49.9 nmol/L, 50.0–74.9 nmol/L, and ≥75.0 nmol/L [21]. Among the individuals with NAFLD included, the percentages of missing data on covariates were lower than 1% (education level (0.09%), smoking status (0.09%), BMI (0.8%), CVD (0.02%), and steroid use (0.07%)) except for family-income-to-poverty ratio (8.2%). Missing values for PIR were assigned to a separate “Unknown” category. No imputation method was used for any variable.

The results of the baseline characteristics were presented as weighted means with standard errors (continuous variables) and frequency with weighted percentages (categorical variables). ANOVA for continuous variables and chi-square test for categorical variables were used to compare differences among groups. Weighted Cox proportional hazards models were developed to estimate the association between serum 25(OH)D levels and mortality.

We set the severe deficiency group as the reference category. The median of each category was applied for testing linear trends. We also treated log-transformed serum 25(OH)D levels as a continuous variable. Apart from the unadjusted model, we adjusted potential covariates progressively in the following three models: Model 1 (sex, age, and race/ethnicity), Model 2 (Model 1 plus education level, PIR, smoking status, physical activity, and BMI), and Model 3 (the fully adjusted model; Model 2 plus hypertension, diabetes, hyperlipidemia, CVD, eGFR, and steroid use). The Hochberg step-up procedure was used to adjust the 95% confidence intervals (CIs) of the hazard ratios (HRs) from Model 3 for multiple testing [22].

We also performed subgroup analyses by age (≥60 or <60 years old), sex, race/ethnicity (White or other), smoking status (never or former and current), BMI (≥30 or <30 kg/m^2^), physical activity (sedentary and insufficient or moderate and high), and eGFR (≤90 or >90 mL/min/1.73 m^2^) and examined the significance of multiplicative interaction terms between the stratification variables and serum 25(OH)D by the Wald test.

We conducted a few sensitivity studies based on the fully adjusted model to examine the robustness of our findings. Firstly, considering the possible seasonal variation in serum 25(OH)D due to sun exposure, we further adjusted for the examination period (1 November through 30 April or 1 May through 31 October) [23]. Secondly, we additionally adjusted for the Healthy Eating Index-2015 (in tertiles), accounting for the possible effect of diet [24]. Thirdly, we excluded deaths during the first 2 years of follow-up to reduce the possibility of reverse causality bias. Fourthly, we excluded non-Hispanic Black individuals for a lower prevalence of NAFLD and a lower diagnostic accuracy of the USFLI [14]. Fifthly, we excluded participants with missing data on their family-income-to-poverty ratio. Lastly, we conducted repeated analyses based on quartiles of serum 25(OH)D levels in the total participants and different ethnic groups given the ethnic disparities in vitamin D levels [25].

All analyses were conducted with the “survey” package in R 4.1.3 (R Foundation for Statistical Computing, Vienna, Austria). Statistical significance was determined by a two-tailed *p* < 0.05.

## 4. Results

The overall weighted mean age and female percentage of the included 4512 individuals were 54.12 years and 43.23%, respectively (Table 1). About one-third (weighted percentage: 33.4%) of individuals were in the severe to moderate deficiency group (<50.0 nmol/L), and about a quarter of individuals (weighted percentage: 25.1%) were in the sufficiency group (≥75.0 nmol/L). Individuals with NAFLD who had higher serum 25(OH)D levels were older; had a greater proportion of non-Hispanic Whites, smokers, and individuals without obesity; and had higher levels of education, family income, and physical activity. We also compared the characteristics of individuals with and without NAFLD (Supplementary Table S3). Compared with individuals without NAFLD, NAFLD patients were more likely to be older, male, non-Hispanic Black, and non-smokers and had less education, family income, and physical activity, with a higher incidence of diabetes, hypertension, hyperlipidemia, and CVD. Individuals with NAFLD also had lower serum 25(OH)D concentrations than those without NAFLD.

**Figure 2:**
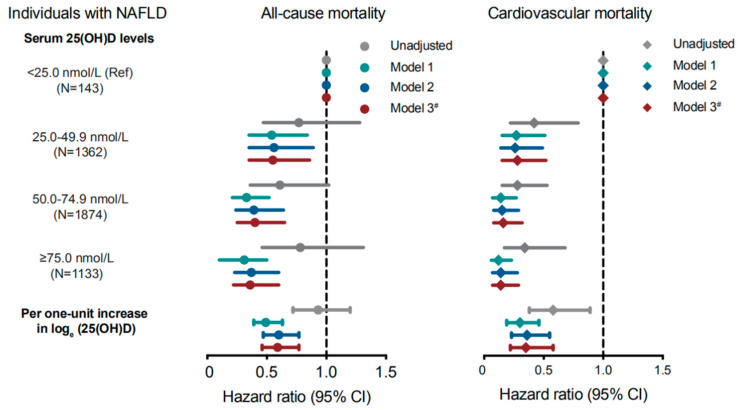
HRs (95% CIs) for all-cause and cardiovascular mortality based on serum 25(OH)D levels among NAFLD patients in NHANES, 2001–2016. HRs are shown as dots and cyan diamonds and 95% CIs as horizontal lines for all-cause and cardiovascular mortality, respectively. Model 1: adjusted for age, sex, and race/ethnicity. Model 2: further adjusted for all covariates in Model 1 and education level, PIR, smoking status, physical activity, and BMI. Model 3 (fully adjusted model): further adjusted for all covariates in Model 2 and hypertension, diabetes, hyperlipidemia, CVD, eGFR, and steroid use. ^#^ All 95% CIs from Model 3 were adjusted using Hochberg step-up procedure. Abbreviations: HRs, hazard ratios; CIs, confidence intervals; NAFLD, nonalcoholic fatty liver disease; NHANES, National Health and Nutrition Examination Survey; PIR, family poverty income ratio, BMI, body mass index; CVD, cardiovascular disease; eGFR, estimated glomerular filtration rate.

In total, 898 all-cause deaths and 305 CVD deaths were documented in NAFLD patients over a median follow-up of 8.7 years. Compared with individuals in the severe deficiency group (<25.0 nmol/L), the multivariable-adjusted HRs and 95% CIs were 0.55 (0.35, 0.86), 0.40 (0.25, 0.65) and 0.36 (0.22, 0.60), respectively, for all-cause mortality (P for trend <0.001); 0.28 (0.15, 0.52), 0.16 (0.08, 0.32), and 0.14 (0.07, 0.29), respectively, for cardiovascular mortality (*p* for trend < 0.001) in the other three groups from lower to higher serum 25(OH)D categories (25.0–49.9, 50.0–74.9, and ≥75.0 nmol/L) in the fully adjusted model (Figure 2). Each 1-unit increase in the natural log-transformed serum 25(OH)D concentration was related to a 41% (HR = 0.59, 95% CI: 0.46, 0.77) lower risk of all-cause mortality and a 65% lower risk (HR = 0.35, 95% CI: 0.22, 0.58) of cardiovascular mortality in the fully adjusted model. We also repeated the analyses among individuals without NAFLD. The impacts of the highest serum 25(OH)D group (≥75.0 nmol/L) compared with the lowest group(<25.0 nmol/L) on all-cause (HR = 0.50, 95% CI: 0.24–1.07) and cardiovascular mortality (HR = 0.38, 95% CI: 0.15–1.01) were insignificant in the fully adjusted model (Supplementary Figure S1).

We observed similar findings in the subgroup analyses (Table 2). No significant interaction effects were found between serum 25(OH)D and the grouping factors except for age (*p* interaction = 0.014).

The results remained consistent after further adjusting for the time when blood was drawn or Healthy Eating Index-2015 based on the fully adjusted model. The findings did not change materially after excluding non-Hispanic Black individuals, individuals who died in the first 2 follow-up years, and individuals with unknown data on PIR (Supplementary Table S4). Similar results were also observed after we categorized serum 25(OH)D levels into quartiles in the total participants and non-Hispanic Whites, whereas insignificant associations were found in other ethnicities (Supplementary Table S5).

## 5. Discussion

Using NHANES 2001–2016 data, we demonstrated that increased serum 25(OH)D concentrations were significantly related to decreased mortality from all causes and cardiovascular diseases in NAFLD patients after adjusting for covariates. The associations were stronger than those in individuals without NAFLD. Subgroup analyses suggested that the relationships in different subgroups were mostly consistent with the total NAFLD patients.

Although numerous previous studies have reported that serum 25(OH)D levels are negatively related to incident NAFLD [26,27,28], only a single study has investigated the effect of serum 25(OH)D on the mortality of NAFLD patients [29]. Kim et al. found that vitamin D deficiency increased mortality from diabetes and Alzheimer’s disease in individuals with NAFLD from the third NHANES survey (1988–1994), but the trend did not reach statistical significance in all-cause or cardiovascular mortality [29]. The inconsistent results between this previous study and our study were probably due to the discrepancies in the characteristics of the study populations, the definition of NAFLD, the sample size, the statistical analysis, and the adjustment for covariates.

Numerous studies have shown that vitamin D protects against the progression and severity of NAFLD, which may support our findings. A retrospective cohort study in South Korea has reported that serum 25(OH)D exhibited a positive linear correlation with the resolution of NAFLD. Maintaining a level of 50.0 nmol/L or above showed an effective approach for reversing the disease course in patients with NAFLD at baseline [30]. Dasarathy et al. found that a low vitamin D level can independently predict the severity of NAFLD [31]. Some clinical trials also demonstrated that vitamin D supplementation has therapeutic effects on patients with NAFLD, including a reduction in liver enzymes, weight, and BMI and an improvement in lipid profile, glycemic control, insulin resistance, and hepatic steatosis and fibrosis [32,33,34,35]. However, a few studies reported insignificant relationships between vitamin D status and histological severity of NAFLD [36]. On the other hand, although there has been accumulating evidence on the inverse associations between serum 25(OH)D levels and death from epidemiological studies among different populations [23,37,38], a meta-analysis of randomized controlled trials indicated no significant impact of vitamin D supplementation on all-cause and cardiovascular deaths [39]. The influence of vitamin D status and supplement use on mortality among NAFLD patients requires further investigation.

There is a lack of understanding of the biological mechanisms behind serum 25(OH)D and NAFLD prognosis. Many animal studies have suggested that vitamin D may protect against NAFLD pathogenesis and progression through several functions, including anti-inflammatory [40,41], anti-oxidant [42], anti-fibrosis [43,44], modulating intra-hepatic lipid accumulation [45], improving insulin sensitivity [46], preserving the gut homeostasis [47], and increasing intestinal absorption of bile acids [48]. Further research is required to clarify how vitamin D affects all-cause and cardiovascular deaths in NAFLD patients.

Interestingly, serum 25(OH)D levels and age group exhibited a significant interaction effect in our subgroup analyses. Higher serum 25(OH)D levels had a more evident protective effect on NAFLD patients aged ≥60 years than on those aged <60 years. Because there have been no studies for reference, we can only speculate about the reasons for this discrepancy. First, the older participants had higher levels of serum 25(OH)D than participants aged <60 in our study, which was in line with previous NHANES studies [23,38]. Secondly, compared with younger individuals, older individuals tend to have more chronic comorbidities, most of which are related to serum 25(OH)D deficiency [6]. Hence, older adults could benefit more from increased serum 25(OH)D than those who are younger. Owing to the decreased sample size in the subgroup analyses, our findings should be interpreted cautiously and need to be further explored in larger populations. On the other hand, we observed insignificant associations in non-White ethnicities based on the quartiles of serum 25(OH)D concentrations. Given the limited number of participants and the randomness of reference values set by quartiles in these ethnic groups, we urged for further confirmation of the results and exploration of vitamin D reference values in specific ethnic populations.

The advantages of our study include rigorous protocols and quality controls, a nationally representative sample, a relatively long follow-up period, and high-quality data from the NHANES study. However, several limitations should be mentioned. First, serum 25(OH)D concentrations were collected at enrollment without repeat measurements, thus the time-varying association has been uncertain. Second, NAFLD was diagnosed by USFLI, which was calculated from a few biochemical indexes rather than by liver biopsy. We were also unable to exclude some other etiologies of fatty liver disease due to insufficient data, such as iron overload, autoimmune hepatitis, drug-induced liver injury, and genetic factors, which may induce diagnostic ascertainment bias. Third, some residual confounders, such as inflammatory factors, exposure to sunlight, and vitamin D intake from dietary sources as well as supplements were unable to be adjusted in our study because data on these issues are only available in specific survey cycles. Moreover, although the blood drawing period was further adjusted in the sensitivity analysis, the seasonality of vitamin D levels may not be fully adjusted due to a lack of a specific month or date of measurement. Last, all participants included were US residents, leading to a limitation in the generalizability of the conclusions to other populations with different socioeconomic characteristics.

## 6. Conclusions

In summary, increasing levels of serum 25(OH)D can benefit NAFLD patients by lowering all-cause and cardiovascular deaths among US adults. The causal relationship needs to be validated in more large-scale prospective studies. Moreover, more well-designed clinical trials are strongly encouraged to explore whether vitamin D supplements from sun exposure, diet, or supplemental use are favorable in the secondary prevention of NAFLD patients. 

## Figures and Tables

**Table 1:** Baseline characteristics of individuals with NAFLD based on serum 25(OH)D levels in NHANES, 2001–2016.

		Serum 25(OH)D Levels (nmol/L)				
Characteristics	Total (N = 4512)	<25.0 (N = 143)	25.0–49.9 (N = 1362)	50.0–74.9 (N = 1874)	≥75.0 (N = 1133)	*p* Value
Age (years)	54.12 ± 0.29	47.52 ± 1.70	50.22 ± 0.60	53.23 ± 0.47	59.37 ± 0.57	<0.001
Sex, n (%)						<0.001
Male	2452 (56.77)	55 (35.06)	652 (48.99)	1112 (62.42)	633 (57.09)	
Female	2060 (43.23)	88 (64.94)	710 (51.01)	762 (37.58)	500 (42.91)	
Race/ethnicity, n (%)						<0.001
Non-Hispanic White	2189 (72.03)	23 (27.87)	435 (55.68)	972 (75.19)	759 (85.32)	
Non-Hispanic Black	508 (6.55)	65 (38.56)	254 (13.62)	127 (3.49)	62 (2.27)	
Mexican American	1042 (10.21)	34 (20.91)	418 (15.67)	470 (10.91)	120 (3.54)	
Others	773 (11.20)	21 (12.66)	255 (15.04)	305 (10.41)	192 (8.87)	
Smoking status, n (%)						<0.001
Never	2495 (55.06)	87 (59.26)	792 (59.22)	1025 (55.18)	591 (51.03)	
Former	1428 (31.40)	26 (18.89)	359 (23.44)	625 (32.77)	418 (37.46)	
Current	585 (13.47)	30 (21.85)	210 (17.34)	222 (12.05)	123 (11.51)	
Education level, n (%)						0.02
Less than high school	1466 (21.53)	46 (30.19)	497 (23.70)	630 (21.65)	293 (18.79)	
High school or equivalent	1023 (24.19)	31 (20.31)	298 (26.30)	410 (22.93)	284 (24.53)	
College or above	2019 (54.23)	66 (49.50)	565 (50.00)	833 (55.42)	555 (56.68)	
Family poverty income ratio, n (%)						<0.001
<1.0	865 (12.62)	35 (23.72)	295 (16.67)	363 (12.06)	172 (9.00)	
≥1.0 and <3.0	1825 (35.72)	67 (47.21)	583 (39.17)	735 (34.65)	440 (33.34)	
≥3.0	1453 (44.97)	29 (20.93)	364 (36.83)	628 (47.05)	432 (51.01)	
Unknown	369 (6.68)	12 (8.14)	120 (7.33)	148 (6.24)	89 (6.65)	
Physical activity, n (%)						<0.001
Sedentary	1479 (28.28)	59 (43.41)	523 (35.33)	546 (25.08)	351 (25.55)	
Insufficient	913 (21.07)	19 (12.06)	272 (21.01)	426 (23.46)	196 (18.35)	
Moderate	527 (12.68)	13 (9.34)	134 (10.82)	240 (13.78)	140 (12.96)	
High	1593 (37.97)	52 (35.19)	433 (32.83)	662 (37.68)	446 (43.15)	
BMI (kg/m^2^), n (%)						<0.001
<25.0	291 (5.06)	6 (6.66)	73 (3.44)	114 (4.69)	98 (6.99)	
25.0–29.9	1346 (28.18)	20 (14.38)	325 (20.92)	603 (29.04)	398 (34.94)	
≥30.0	2839 (66.22)	117 (78.97)	954 (75.64)	1141 (66.27)	627 (58.07)	
Diabetes, n (%)	1526 (28.31)	50 (30.60)	506 (32.22)	594 (26.11)	376 (27.91)	0.05
Hypertension, n (%)	2745 (58.58)	85 (56.77)	782 (54.51)	1084 (55.49)	794 (66.81)	<0.001
Hyperlipidemia, n (%)	3970 (88.12)	121 (81.51)	1169 (87.00)	1664 (88.73)	1016 (88.72)	0.21
CVD, n (%)	815 (16.18)	28 (19.79)	233 (15.32)	305 (14.63)	249 (18.92)	0.03
Steroid use, n (%)	84 (1.57)	3 (2.31)	24 (1.51)	32 (1.52)	25 (1.63)	0.93
eGFR (mL/min/1.73 m^2^)	88.32 ± 0.42	97.75 ± 2.92	94.83 ± 0.81	89.44 ± 0.61	80.20 ± 0.62	<0.001
Waist circumference (cm)	112.12 ± 0.32	115.67 ± 1.97	114.70 ± 0.50	111.85 ± 0.52	109.97 ± 0.55	<0.001
Total cholesterol (mg/dL)	195.48 ± 0.89	187.47 ± 3.45	196.97 ± 1.74	195.59 ± 1.22	194.63 ± 1.56	0.12
Low-density lipoprotein cholesterol (mg/dL)	115.43 ± 0.74	113.31 ± 2.96	117.26 ± 1.55	115.82 ± 1.08	113.45 ± 1.19	0.18
High-density lipoprotein cholesterol (mg/dL)	46.52 ± 0.21	46.94 ± 0.92	45.47 ± 0.42	45.53 ± 0.29	48.84 ± 0.49	<0.001
Fasting triglyceride (mg/dL)	173.24 ± 2.91	137.76 ± 8.70	177.16 ± 6.82	177.85 ± 3.73	165.88 ± 4.43	<0.001
Fasting glucose (mg/dL)	118.74 ± 0.77	126.71 ± 5.93	123.00 ± 1.79	117.48 ± 1.01	116.19 ± 1.08	0.01
Fasting insulin (pmol/L)	20.87 ± 0.35	24.83 ± 1.25	23.00 ± 0.56	20.88 ± 0.63	18.65 ± 0.50	<0.001
Glycosylated hemoglobin, Type A1C (%)	5.99 ± 0.02	6.22 ± 0.14	6.13 ± 0.05	5.93 ± 0.03	5.95 ± 0.04	0.001
Gamma-glutamyltransferase (U/L)	36.98 ± 0.61	41.16 ± 5.47	36.37 ± 1.10	36.02 ± 0.83	38.59 ± 1.39	0.31

Data are presented as weighted means ± SEs for continuous variables and unweighted numbers (weighted percentages) for categorical variables. Abbreviations: NAFLD, nonalcoholic fatty liver disease; NHANES, National Health and Nutrition Examination Survey; BMI, body mass index; CVD, cardiovascular disease; eGFR, estimated glomerular filtration rate.

**Table 2:** Subgroup analyses of the associations (HRs, 95% CIs) between serum 25(OH)D levels and all-cause mortality among individuals with NAFLD in NHANES, 2001–2016.

		Serum 25(OH)D Levels (nmol/L)				
Subgroup	No. Deaths/Total	<25.0	25.0–49.9	50.0–74.9	≥75.0	*p* for Interaction
Age, years						0.014
<60	126/2266	1.00	0.87 (0.43, 1.78)	0.42 (0.18, 0.99)	0.34 (0.13, 0.90)	
≥60	772/2246	1.00	0.47 (0.28, 0.79)	0.39 (0.22, 0.68)	0.35 (0.20, 0.62)	
Sex						0.568
Male	554/2452	1.00	0.78 (0.31, 1.93)	0.50 (0.20, 1.23)	0.50 (0.20, 1.27)	
Female	344/2060	1.00	0.46 (0.25, 0.86)	0.40 (0.21, 0.75)	0.31 (0.16, 0.60)	
Race/ethnicity						0.186
White	604/2189	1.00	0.51 (0.27, 0.95)	0.36 (0.19, 0.69)	0.31 (0.16, 0.58)	
Non-White	294/2323	1.00	0.59 (0.31, 1.12)	0.41 (0.22, 0.75)	0.53 (0.27, 1.04)	
Smoking status						0.770
Never	367/2495	1.00	0.58 (0.34, 1.00)	0.38 (0.22, 0.64)	0.35 (0.21, 0.60)	
Former/Current	528/2013	1.00	0.48 (0.21, 1.09)	0.35 (0.16, 0.78)	0.32 (0.14, 0.73)	
BMI, kg/m^2^						0.300
<30	396/1637	1.00	2.53 (0.50, 12.68)	1.76 (0.35, 8.73)	1.49 (0.29, 7.55)	
≥30	475/2839	1.00	0.41 (0.24, 0.71)	0.30 (0.17, 0.52)	0.28 (0.17, 0.47)	
Physical activity						0.723
Sedentary/Insufficient	608/2329	1.00	0.50 (0.31, 0.81)	0.37 (0.22, 0.61)	0.34 (0.20, 0.56)	
Moderate/High	290/2120	1.00	0.58 (0.20, 1.69)	0.33 (0.11, 0.98)	0.34 (0.12, 0.98)	
eGFR, mL/min/1.73 m^2^						0.222
≤90	742/2358	1.00	0.41 (0.24, 0.69)	0.35 (0.20, 0.59)	0.31 (0.19, 0.51)	
>90	156/2154	1.00	1.05 (0.37, 2.99)	0.62 (0.22, 1.77)	0.71 (0.24, 2.14)	

All the models were adjusted for age, sex, race/ethnicity, education level, PIR, smoking status, physical activity, BMI, hypertension, diabetes, hyperlipidemia, CVD, eGFR and steroid use, with exception of stratifying factors. Abbreviations: HRs, hazard ratios; CIs, confidence intervals; NAFLD, nonalcoholic fatty liver disease; NHANES, National Health and Nutrition Examination Survey; PIR, family poverty income ratio; BMI, body mass index; CVD, cardiovascular disease; eGFR, estimated glomerular filtration rate.

## Data Availability

This study used openly available datasets at https://www.cdc.gov/nchs/nhanes/index.htm (accessed on 1 June 2022).

## Supplementary Materials

The following are available online at https://www.mdpi.com/article/10.3390/nu14194013/s1, Table S1: Methods and regression equations for plasm glucose in NHANES cycles, 2001–2016, Table S2: Methods and regression equations for serum insulin in NHANES cycles, 2001–2016, Table S3: Baseline characteristics of individuals based on the presence of NAFLD in NHANES, 2001–2016, Table S4: Sensitivity analyses of the associations (HRs, 95% CIs) between serum 25(OH)D concentrations and all-cause and cardiovascular mortality among patients with NAFLD in NHANES, 2001–2016, Table S5: Sensitivity analysis of the associations (HRs, 95% CIs) between serum 25(OH)D concentrations and all-cause and cardiovascular mortality among patients with NAFLD of different ethnic groups in NHANES, 2001–2016, Figure S1: HRs (95% CIs) for all-cause and cardiovascular mortality based on serum 25(OH)D levels among individuals without NAFLD in NHANES, 2001–2016.
