# Supplementary material for: Higher Serum 25-Hydroxyvitamin D Is Associated with Lower All-Cause and Cardiovascular Mortality among US Adults with Nonalcoholic Fatty Liver Disease
Source: Nutrients. 2022 Sep 27;14(19):4013. doi: 10.3390/nu14194013 (PMC9571761; doi:10.3390/nu14194013)
Supplement: Supplementary file 1 [file nutrients-14-04013-s001.zip › nutrients-1909792-supplementary.pdf]

Supplementary Table S1. Methods and regression equations for plasm glucose in NHANES cycles, 2001-2016

Survey cycles	Method	Backward regression equations
2015-2016	Hexokinase method using a Roche/Hitachi Cobas C311 Chemistry Analyzer (Roche Diagnostics, Indianapolis, IN), University of Missouri-Columbia, Columbia, MO [1]	To compare 2015-2016 data (X) and 2013-2014 data (Y): $Y=1.023*X-0.5108$
2013-2014	Hexokinase method using a Roche/Hitachi Cobas C501 Chemistry Analyzer (Roche Diagnostics, Indianapolis, IN), University of Missouri-Columbia, Columbia, MO [2]	Assumed comparable to 2007-2012 data
2007-2012	Hexokinase method using a Roche/Hitachi Modular P Chemistry Analyzer (Roche Diagnostics, Indianapolis, IN), University of Minnesota, Minneapolis, MN [3]	To compare 2007-2014 data (X) and 2005-2006 data (Y): $Y=X-1.139$
2005-2006	Hexokinase method using a Roche/Hitachi 911 Analyzer (Roche Diagnostics, Indianapolis, IN), University of Minnesota, Minneapolis, MN [4]	To compare 2005-2006 data (X) and 2001-2004 data (Y): $Y=0.9835*X$
2001-2004	Modified hexokinase enzymatic method using a Roche Cobas Mira Chemistry System (Roche Diagnostic Systems, Inc., Montclair, NJ), University of Missouri-Columbia, Columbia, MO [5]	-

Supplementary Table S2. Methods and regression equations for serum insulin in NHANES cycles, 2001-2016

Survey cycles	Method	Backward regression equations
2013-2016	Two-site immunoenzymometric assay, AIA-PACK IRI (Tosoh AIA-900 Chemistry Analyzer, Inc., San Francisco, CA), University of Missouri-Columbia, Columbia, MO [6]	To compare 2013-2016 data (X) and 2009-2012 data (Y): Y=10^(0.9765*log10(X) + 0.07832)
2011-2012	Chemiluminescent immunoassay, Elecsys 2010 analyzer (Roche Diagnostics, Indianapolis, IN), University of Minnesota, Minneapolis, MN [7]	To compare 2011-2012 data (X) and 2005-2010 data (Y): Y=0.6295+1.0770*X–0.0008566*X^2
2005-2010	Merocodia Insulin ELISA (Merocodia AB, Uppsala, Sweden), University of Minnesota, Minneapolis, MN [8]	To compare 2005-2010 data (X) and 2003-2004 data (Y): Y=1.0526*X–1.5674
2003-2004	Two-site immunoenzymometric assay, AIA-PACK IRI (Tosoh Medics, Inc., San Francisco, CA), University of Missouri-Columbia, Columbia, MO [9]	To compare 2003-2004 (X) and 2001-2002 data (Y): Y=0.96006*X+3.23663
2001-2002	Radioimmunoassay, Berthold Models LB2111 and LB2104 Multi-Crystal Gamma Counter (Wallac Inc., Gaithersburg, MD), University of Missouri-Columbia, Columbia, MO [10]	-

References

1. NCHS. NHANES 2015-2016 Laboratory Procedure Manual - Fasting Glucose. Accessed June 2022. https://wwwn.cdc.gov/nchs/data/nhanes/2015-2016/labmethods/GLU_I_MET_C311.pdf
2. NCHS. NHANES 2013-2014 Laboratory Procedure Manual - Fasting Glucose. Accessed June 2022. https://wwwn.cdc.gov/nchs/data/nhanes/2013-2014/labmethods/GLU_H_MET_Fasting_Glucose.pdf
3. NCHS. NHANES 2007-2008 Laboratory Procedure Manual - Fasting Glucose. Accessed June 2022. http://www.cdc.gov/nchs/data/nhanes/nhanes_07_08/glu_e_met_fasting_glucose_ModP.pdf

4. NCHS. NHANES 2005-2006 Laboratory Procedure Manual - Plasma Fasting Glucose. Accessed June 2022. http://www.cdc.gov/nchs/data/nhanes/nhanes_05_06/glu_d_met_fasting_glucose.pdf
5. NCHS. NHANES 2003-2004 Laboratory Procedure Manual - Plasma Fasting Glucose. Accessed June 2022. https://www.cdc.gov/nchs/data/nhanes/nhanes_03_04/l10am_c_met_glucose.pdf
6. NCHS. NHANES 2015-2016 Laboratory Procedure Manual - Insulin. Accessed June 2022. https://wwwn.cdc.gov/nchs/data/nhanes/2015-2016/labmethods/INS_I_MET.pdf
7. NCHS. NHANES 2009-2010 Laboratory Procedure Manual - Insulin. Accessed June 2022. http://www.cdc.gov/NCHS/data/nhanes/nhanes_09_10/INS_F_met_insulin.pdf
8. NCHS. NHANES 2005-2006 Laboratory Procedure Manual - Insulin. Accessed June 2022. http://www.cdc.gov/nchs/data/nhanes/nhanes_05_06/glu_d_met_insulin.pdf
9. NCHS. NHANES 2003-2004 Laboratory Procedure Manual - Lab 10AM - Insulin. Accessed June 2022. http://www.cdc.gov/nchs/data/nhanes/nhanes_03_04/l10am_c_met_insulin.pdf
10. NCHS. NHANES 1999-2000 Laboratory Procedure Manual - Lab 10AM - Insulin. Accessed June 2022. http://www.cdc.gov/nchs/data/nhanes/nhanes_99_00/lab10am_met_insulin.pdf

Supplementary Table S3. Baseline characteristics of individuals based on the presence of NAFLD in NHANES, 2001–2016

Characteristics	Total (N =11,029)	NAFLD (N =4512)	No NAFLD (N =6517)	*P* value
Age (years)	50.27±0.29	54.12±0.29	47.83±0.37	<0.001
Sex, n (%)				<0.001
Male	5379 (50.91)	2452 (56.77)	2927 (44.21)	
Female	5650 (49.09)	2060 (43.23)	3590 (55.79)	
Race/ethnicity, n (%)				<0.001
Non-Hispanic White	5199 (69.44)	2189 (72.03)	3010 (67.80)	
Non-Hispanic Black	2117 (11.21)	508 (6.55)	1609 (14.16)	
Mexican American	1730 (7.04)	1042 (10.21)	688 (5.03)	
Others	1983 (12.31)	773 (11.20)	1210 (13.01)	
Smoking status, n (%)				<0.001
Never	6527 (59.83)	2495 (55.06)	4032 (62.89)	
Former	2897 (25.41)	1428 (31.40)	1469 (21.63)	
Current	1595 (14.69)	585 (13.47)	1010 (15.47)	
Education level, n (%)				<0.001
Less than high school	2934 (17.63)	1466 (21.53)	1468 (15.16)	
High school or equivalent	2437 (22.52)	1023 (24.19)	1414 (21.46)	
College or above	5649 (59.82)	2019 (54.23)	3630 (63.38)	
Family poverty income ratio, n (%)				<0.001
<1.0	1945 (12.21)	865 (12.62)	1080 (11.96)	
≥1.0 & <3.0	4246 (33.33)	1825 (35.72)	2421 (31.81)	
≥3.0	3996 (48.15)	1453 (44.97)	2543 (50.17)	
Unknown	842 (6.31)	369 (6.68)	473 (6.06)	
Physical activity, n (%)				<0.001

Sedentary	3066 (23.04)	1479 (28.28)	1587 (19.72)	<0.001
Insufficient	2345 (22.45)	913 (21.07)	1432 (23.32)	
Moderate	1384 (13.28)	527 (12.68)	857 (13.66)	
High	4234 (41.23)	1593 (37.97)	2641 (43.30)	
BMI (kg/m^2), n (%)				<0.001
<25.0	3221 (30.09)	291 (5.06)	2930 (46.05)	
25.0–29.9	3727 (33.64)	1346 (28.18)	2381 (37.16)	
≥30.0	4026 (35.69)	2839 (66.22)	1187 (16.79)	
Diabetes, n (%)	2116 (14.77)	1526 (28.31)	590 (6.18)	<0.001
Hypertension, n (%)	5161 (41.34)	2745 (58.58)	2416 (30.40)	<0.001
Hyperlipidemia, n (%)	8344 (74.41)	3970 (88.12)	4374 (65.72)	<0.001
CVD, n (%)	1435 (10.62)	815 (16.18)	620 (7.10)	0.03
Steroid use, n (%)	172 (1.44)	84 (1.57)	88 (1.36)	0.43
eGFR (mL/min/1.73 m^2)	91.86±0.38	88.32±0.42	94.11±0.48	<0.001
Waist circumference (cm)	99.27±0.25	112.12±0.32	91.12±0.22	<0.001
Total cholesterol (mg/dL)	195.04±0.54	195.48±0.89	194.76±0.67	0.51
Low-density lipoprotein cholesterol (mg/dL)	115.97±0.45	115.43±0.74	116.30±0.55	0.34
High-density lipoprotein cholesterol; (mg/dL)	53.21±0.21	46.52±0.21	57.46±0.27	<0.001
Fasting triglyceride (mg/dL)	132.10±1.40	173.24±2.91	106.00±1.33	<0.001
Fasting glucose (mg/dL)	105.98±0.38	118.74±0.77	97.89±0.27	<0.001
Fasting insulin (pmol/L)	12.63±0.18	20.87±0.35	7.40±0.07	<0.001
Glycosylated Hemoglobin, Type A1C (%)	5.65±0.01	5.99±0.02	5.43±0.01	<0.001
Gamma-glutamyltransferase (U/L)	25.66±0.27	36.98±0.61	18.47±0.16	<0.001
25(OH)D (nmol/L)	67.70±0.54	64.35±0.67	69.83±0.63	<0.001

Data are presented as weighted means ± SEs for continuous variables and unweighted numbers (weighted percentages) for categorical variables.

Abbreviations: NAFLD, nonalcoholic fatty liver disease; NHANES, National Health and Nutrition Examination Survey; BMI, body mass index; CVD, cardiovascular disease; eGFR, estimated glomerular filtration rate.

Supplementary Table S4. Sensitivity analyses of the associations (HRs, 95% CIs) between serum 25(OH)D concentrations and all-cause and cardiovascular mortality among patients with NAFLD in NHANES, 2001–2016

Analysis	Serum 25(OH)D concentrations (nmol/l)				*P* trend
	<25.0	25.0–49.9	50.0–74.9	≥75.0	
Further adjustment of months when blood was drawn (N=4512)					
All-cause mortality	1.00	0.56 (0.36, 0.87)	0.40 (0.25, 0.64)	0.37 (0.23, 0.59)	<0.001
Cardiovascular mortality	1.00	0.28 (0.15, 0.50)	0.16 (0.09, 0.31)	0.14 (0.07, 0.28)	<0.001
Further adjustment of HEI-2015 (in tertiles) (N=3958)					
All-cause mortality	1.00	0.51 (0.32, 0.81)	0.38 (0.23, 0.62)	0.32 (0.20, 0.53)	<0.001
Cardiovascular mortality	1.00	0.24 (0.13, 0.45)	0.17 (0.09, 0.33)	0.14 (0.07, 0.28)	<0.001
Excluding non-Hispanic Black participants (N=4004)					
All-cause mortality	1.00	0.54 (0.32, 0.91)	0.39 (0.22, 0.66)	0.35 (0.20, 0.60)	<0.001
Cardiovascular mortality	1.00	0.24 (0.13, 0.46)	0.14 (0.07, 0.29)	0.13 (0.06, 0.26)	<0.001
Excluding participants who died within two years of follow-up (N=4406)					
All-cause mortality	1.00	0.53 (0.33, 0.86)	0.37 (0.22, 0.61)	0.36 (0.22, 0.60)	0.004
Cardiovascular mortality	1.00	0.30 (0.15, 0.57)	0.15 (0.07, 0.31)	0.13 (0.06, 0.29)	<0.001
Excluding participants with missing data on family income to poverty ratio (N=4143)					
All-cause mortality	1.00	0.56 (0.35, 0.92)	0.40 (0.24, 0.66)	0.37 (0.22, 0.61)	<0.001
Cardiovascular mortality	1.00	0.30 (0.15, 0.60)	0.18 (0.09, 0.37)	0.15 (0.07, 0.32)	<0.001

All the models adjusted for age, sex, race/ethnicity, education level, PIR, smoking status, BMI, physical activity, diabetes, hypertension, dyslipidemia, CVD, eGFR, and steroid use.

Abbreviations: HRs, hazard ratios; CIs, confidence intervals; NAFLD, nonalcoholic fatty liver disease; NHANES, National Health and Nutrition Examination Survey; PIR, family poverty income ratio; BMI, body mass index; CVD, cardiovascular disease; eGFR, estimated glomerular filtration rate.

Supplementary Table S5. Sensitivity analysis of the associations (HRs, 95% CIs) between serum 25(OH)D concentrations and all-cause and cardiovascular mortality among patients with NAFLD of different ethnic groups in NHANES, 2001–2016

	Quartiles of serum 25(OH)D concentrations (nmol/l)				
	<44.7	44.7–59.3	59.4–74.9	≥75.0	*P* trend
Total participants (N=4512)					
All-cause mortality	1.00	0.77 (0.58, 1.03)	0.69 (0.53, 0.89)	0.63 (0.48, 0.82)	0.001
Cardiovascular mortality	1.00	0.55 (0.31, 0.95)	0.47 (0.29, 0.77)	0.41 (0.27, 0.64)	<0.001
Non-Hispanic White (N=2189)	<53.3	53.3-66.1	66.2-80.6	⩾80.7	
All-cause mortality	1.00	0.73 (0.56, 0.95)	0.65 (0.48, 0.88)	0.63 (0.48, 0.84)	0.003
Cardiovascular mortality	1.00	0.48 (0.28, 0.81)	0.64 (0.38, 1.06)	0.49 (0.32, 0.76)	0.012
Non-Hispanic Black (N=508)	<30.5	30.6-42.5	42.6-60.3	⩾60.4	
All-cause mortality	1.00	0.98 (0.46, 2.10)	0.62 (0.29, 1.31)	0.62 (0.32, 1.19)	0.098
Cardiovascular mortality	1.00	0.38 (0.08, 1.71)	0.01 (0, 0.12)	0.41 (0.14, 1.27)	0.083
Other races (N=1815)	<41.9	42.0-54.6	54.7-68.3	⩾68.4	
All-cause mortality	1.00	0.97 (0.49, 1.91)	0.81 (0.44, 1.49)	0.89 (0.50, 1.59)	0.636
Cardiovascular mortality	1.00	1.01 (0.42, 2.43)	0.41 (0.13, 1.28)	0.63 (0.26, 1.49)	0.204

All the models adjusted for age, sex, race/ethnicity, education level, PIR, smoking status, BMI, physical activity, diabetes, hypertension, dyslipidemia, CVD, eGFR, and steroid use.

Abbreviations: HRs, hazard ratios; CIs, confidence intervals; NAFLD, nonalcoholic fatty liver disease; NHANES, National Health and Nutrition Examination Survey; PIR, family poverty income ratio; BMI, body mass index; CVD, cardiovascular disease; eGFR, estimated glomerular filtration rate.

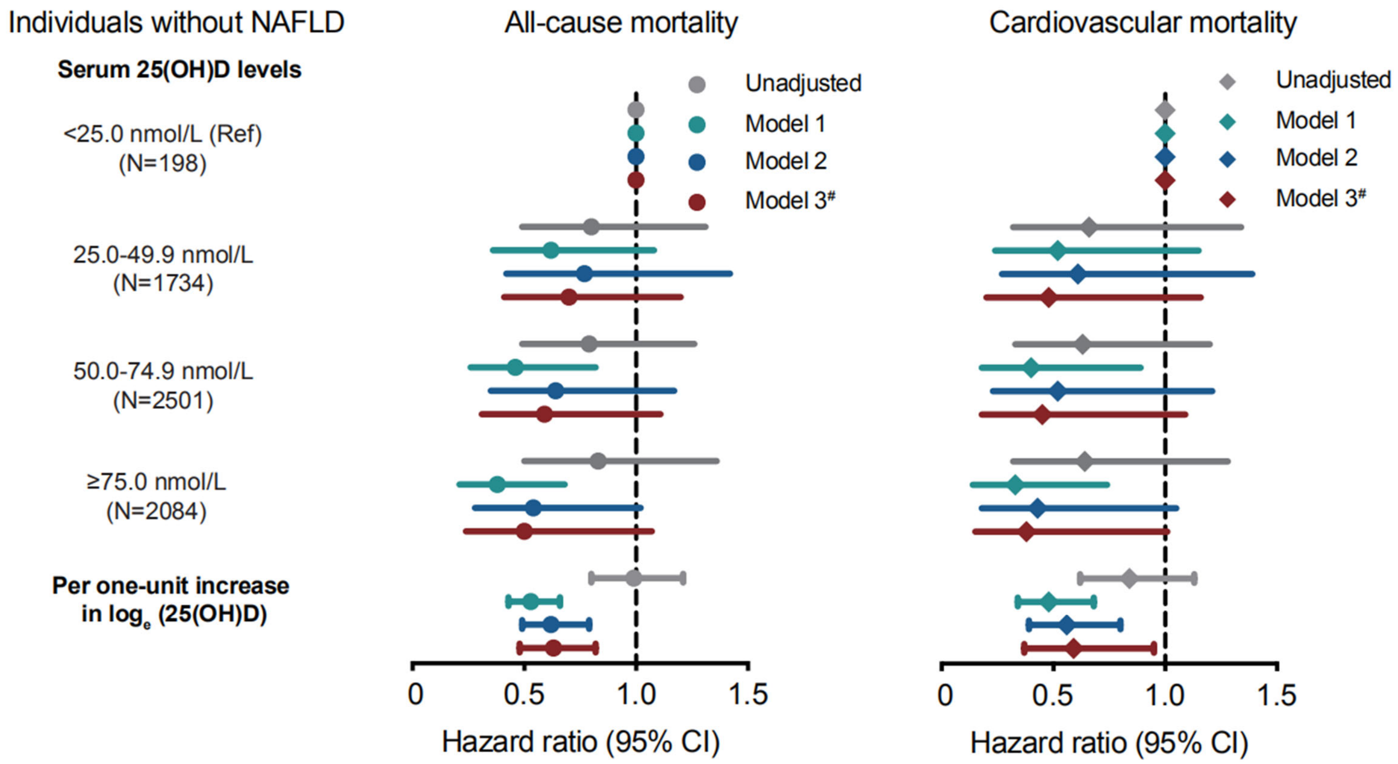

Supplementary Figure S1. HRs (95% CIs) for all-cause and cardiovascular mortality based on serum 25(OH)D levels among individuals without NAFLD in NHANES, 2001–2016.

HRs are shown as dots and cyan diamonds and 95% CIs as horizontal lines for all-cause, and cardiovascular mortality, respectively.

Model 1: adjusted for age, sex, and race/ethnicity.
Model 2: further adjusted for all covariates in Model 1 and education level, PIR, smoking status, physical activity, and BMI.
Model 3 (fully adjusted model): further adjusted for all covariates in Model 2 and hypertension, diabetes, hyperlipidemia, CVD, eGFR, and steroid use.
[#]All the 95% CIs from Model 3 were adjusted using Hochberg step-up procedure.
Abbreviations: HRs, hazard ratios; CIs, confidence intervals; NAFLD, nonalcoholic fatty liver disease; NHANES, National Health and Nutrition Examination Survey; PIR, family poverty income ratio, BMI, body mass index; CVD, cardiovascular disease; eGFR, estimated glomerular filtration rate.
